# Refractive Errors and Amblyopia in Mexican Children Aged 6–12 Years: Clinical Prevalence and Visual Impact

**DOI:** 10.3390/children12121641

**Published:** 2025-12-02

**Authors:** Abraham García-Gil, Israel Gómez-Torales, Kalahary Patricia García-Nahara, Marco Antonio Luna-Ruiz-Esparza, Eduardo Espinoza-Angulo, Héctor Machado-Jiménez, Leticia Riverón-Negrete, Humberto Gómez-Campaña, Abraham Campos-Romero, Jonathan Alcántar-Fernández

**Affiliations:** 1 Innovation and Research Department, Salud Digna, Culiacan 80184, Mexico; abraham.gil@salud-digna.org (A.G.-G.); marco.luna@salud-digna.org (M.A.L.-R.-E.); abraham.campos@salud-digna.org (A.C.-R.); 2 Optometry Department, Salud Digna, Culiacan 80184, Mexico; israel.gomez@salud-digna.org (I.G.-T.); kalahary.garcia@salud-digna.org (K.P.G.-N.); eduardo.espinoza@salud-digna.org (E.E.-A.); hector.machado@salud-digna.org (H.M.-J.); 3 Unidad de Genética de la Nutrición, Instituto de Investigaciones Biomédicas, Universidad Nacional Autónoma de Mexico, Instituto Nacional de Pediatría, Ciudad de Mexico 04530, Mexico; lriveronn@pediatria.gob.mx; 4 Medical Direction, Salud Digna, Culiacan 80184, Mexico; investigacion@salud-digna.org

**Keywords:** refractive errors, myopia, astigmatism, hyperopia, amblyopia, visual impairment

## Abstract

**Highlights:**

**What are the main findings?**

- Myopia is the most clinically prevalent refractive error in Mexican children.
- Astigmatism is the main contributor to refractive amblyopia and related visual impairment in Mexican outpatient children.

**What are the implications of the main findings?**

- It is important to implement and strengthen school-based screening programs for refractive errors and for amblyopia.
- Training initiatives for teachers and school health personnel, along with public awareness campaigns led by optometrists, are essential.

**Abstract:**

**Background:** Refractive errors (REs) and amblyopia are the leading causes of visual impairment (VI) in children worldwide; however, national data for Mexico are scarce. **Objective:** We aim to estimate the clinical prevalence of RE, refractive amblyopia, and associated visual impairment (VI) in Mexican children aged 6–12 years. **Methods:** We analyzed 784,372 non-cycloplegic eye examinations from Salud Digna outpatient clinics across all 32 Mexican states (2021–2025). REs were classified as myopia (≤−0.50 D), hyperopia (≥+2.00 D), or astigmatism (≤ −1.00 D cylinder). Refractive amblyopia was defined as best-corrected visual acuity ≤ 20/30 in the most affected eye. The VI categories followed the WHO criteria. **Results:** Myopia was the most frequent (43.86%), followed by astigmatism (38.41%) and hyperopia (1.29%). Refractive amblyopia affected 4.94% of the children, predominantly due to astigmatic isoametropia (72.19%). VI related to refractive amblyopia occurred in 1.20% (mild), 0.37% (moderate), 0.01% (severe), and 0.01% (blindness) of children. Men showed a higher prevalence of RE and refractive amblyopia (*p* < 0.01). Geographic disparities were evident, with the central and southern states reporting the highest RA rates. **Conclusions:** Our outpatient-based study underscores REs, refractive amblyopia, and astigmatic ametropia as significant public health concerns in Mexican school-aged children. The high prevalence of uncorrected REs and refractive amblyopia highlights the need for nationwide school-based screening programs and early intervention strategies to mitigate long-term visual and developmental consequences.

## 1. Introduction

Globally, refractive errors (REs) are the leading cause of vision impairment (VI) [1]. These ocular conditions arise from a mismatch between the axial length of the eye and its refractive power, resulting in blurred vision [2]. RE are typically classified into three main types: myopia, characterized by excessive dioptric power relative to the axial length, leading to blurred distance vision [2,3] hyperopia, where insufficient dioptric power causes difficulty in focusing on near objects [2,4]; and astigmatism, which results from an irregular corneal curvature, producing distorted images at all distances [2,4].

Early detection and appropriate treatment of REs can significantly reduce the burden of VI. Corrective interventions, such as eyeglasses, contact lenses, and refractive surgery, are effective in restoring visual function [5].

If left untreated, REs can lead to further complications, most notably amblyopia, which is a major public health concern that primarily affects children and young adults. Amblyopia is characterized by reduced visual acuity resulting from abnormal visual processing, despite the absence of structural abnormalities or ocular disease [6,7]. It is classified into four types according to its underlying cause: refractive, strabismic, visual deprivation, and iatrogenic occlusive amblyopia [6]. Among these, refractive amblyopia is the most prevalent in the general population and, if not addressed early, significantly increases the risk of permanent VI [8,9].

Refractive amblyopia develops when uncorrected REs interfere with normal visual neurodevelopment owing to insufficient visual stimulation during early childhood [10]. When an RE disproportionately affects one eye, it can lead to anisometropic amblyopia [11], and the severity of amblyopia tends to increase with the degree of anisometropia [12,13]. Conversely, isoametropic amblyopia may occur when a high-magnitude RE is present in both eyes [11].

The primary goal of amblyopia treatment is to enhance visual and binocular development, which, in turn, supports broader neurocognitive functions such as visual–motor coordination and attention. The earlier the treatment is initiated, the greater the likelihood of successful visual recovery [14]. For children under the age of seven, the most widely recognized intervention involves occlusion therapy, typically using an eye patch over the stronger eye to stimulate the weaker eye. In cases of refractive amblyopia, the first-line treatment consists of the full-time wearing of corrective eyeglasses to address the underlying RE [6,15,16].

The impact of amblyopia extends beyond reduced visual acuity. Affected individuals often exhibit a range of functional deficits, including inaccurate accommodative responses, poor monocular fixation, impaired eye tracking, diminished contrast sensitivity, spatial distortions, and increased susceptibility to contour interaction effects [17,18,19,20,21,22,23,24,25]. Moreover, children with amblyopia may experience compromised motor skills, lower self-perceived peer acceptance and physical competence, and slower reading speeds, all of which can significantly hinder their social development and emotional well-being [26,27,28,29,30,31].

The prevalence estimates for amblyopia vary depending on the definition used, as well as the age and ethnicity of the population studied. In 2019, approximately 99.2 million individuals worldwide lived with amblyopia, with projections suggesting that this number will rise to 221.9 million by 2040 [9]. The global prevalence among children is estimated to be 1.36% [9,32,33,34,35,36,37]. A 2022 study by Hu et al. [32] reported that males are more frequently affected than females. Additionally, several risk factors have been associated with amblyopia development, including premature birth, mental disability, and having a first-degree relative with the condition [37,38,39,40,41].

According to the 2020 Population and Housing Census, 2,691,338 individuals in Mexico have VI [42]. Additional studies estimate that 11.01 million Mexicans (approximately 8.7% of the population) are blind or visually impaired, with RE accounting for 2.61 million cases [43]. The prevalence of amblyopia in the general population is estimated to range between 2% and 5% [44,45]. Although previous studies in Mexico have reported the prevalence of RE among schoolchildren [46,47,48], these investigations were limited by small sample sizes or regional scope and did not analyze amblyogenic ametropia or geographical disparities in detail. Most were school-based screenings, which do not capture outpatient clinical patterns or the burden of refractive amblyopia and associated visual impairment. Furthermore, granular geographic comparisons across all 32 states are scarce and have not analyzed amblyogenic ametropias in detail.

Salud Digna is a non-profit organization that offers diagnostic services across Mexico, managing over 200 outpatient healthcare facilities nationwide. In the domain of eye care services, it constitutes the largest network of optometry services, employing approximately 15% of the national optometry workforce [49] and, since 2015 has been the foremost national provider of eyeglasses. Leveraging eye examination data from Salud Digna, this study aimed to evaluate the national geographic clinical distribution of RE and refractive amblyopia and their impact on visual function across sex and age groups in school-aged children. Our study addressed these gaps by leveraging a large, standardized dataset from a nationwide outpatient network to estimate the clinical prevalence of refractive errors, refractive amblyopia, and related visual impairment while exploring sex-, age-, and region-specific trends. The results lay the groundwork for upcoming studies designed to guide public health strategies focused on alleviating the impact of RE and refractive amblyopia in Mexican children.

## 2. Materials and Methods

### 2.1. Study Population

In this cross-sectional retrospective study, we analyzed anonymized electronic health records from non-cycloplegic eye examinations conducted in children aged 6 to 12 years at Salud Digna outpatient clinics across all 32 Mexican states between 1 January 2021 and 30 June 2025. All examinations were performed by certified optometrists, following a standardized clinical protocol. Briefly, patient identity was verified, and a clinical history questionnaire was completed. The instruments were disinfected before use. Lensometry was performed on patients wearing corrective lenses. Interpupillary and nasopupillary distances were measured, and uncorrected visual acuity was assessed using digital optotypes at a distance of 3 m (CS Pola 600, CS 550, Essilor Instruments, Paris, France). Objective refraction was obtained using an autorefractor (AKR550 and WAM 800; Essilor Instruments, Paris, France), followed by refinement of the prescription and evaluation of distance and near visual acuity. Examination results and recommendations were provided, with referrals to ophthalmology or retinal imaging when indicated. Demographic and clinical data were collected using a validated uniform survey instrument implemented consistently across all facilities.

Data from the right eye were used for the analysis of RE clinical prevalence. REs were defined as follows: myopia, spherical equivalent (SE) ≤ −0.50 diopters (D) [4] hyperopia, SE ≥ +2.00 D [50] and astigmatism, cylinder ≤ −1.00 D [4].

Amblyogenic ametropia, defined as an RE of sufficient magnitude to disrupt normal visual development and induce amblyopia if left uncorrected, was classified according to the Optometric Clinical Practice Guideline: Care of the Patient with Amblyopia [7] as follows:

Anisometropic ametropias.

- Presence of myopia with a difference between the eyes > 3.00 D SE;
- Presence of hyperopia with a difference between eyes > 1.00 D SE;
- Presence of astigmatism with a difference between the eyes of >1.50 D cylinder;
- Isometropic ametropias;
- Presence of astigmatism <−2.50 D cylinder in both eyes;
- Presence of hyperopia > 5.00 D SE in both eyes;
- Presence of myopia <−8.00 D SE in both eyes.

Amblyopia was defined as best-corrected visual acuity (BCVA) worse than or equal to 20/30 in the most affected eye [51]. VI was assessed and categorized based on BCVA, following World Health Organization (WHO) guidelines [52].

### 2.2. Statistical Analysis

Descriptive statistics were generated using Microsoft Power Query and Excel to summarize the demographic and clinical characteristics. Inferential analyses were performed using R software (v4.4.0) [53]. Prevalence estimates for refractive errors (RE), refractive amblyopia, and visual impairment (VI) were computed using the dplyr (v1.1.4) and epitools (v0.5-10.1) packages. Exact 95% confidence intervals (CIs) were obtained using the binom.exact() function in epitools, which applies the Clopper–Pearson method for binomial proportions. Associations between categorical variables were assessed using chi-square tests (chisq.test()). All tests were two-tailed, with statistical significance set at *p* < 0.05.

## 3. Results

Between 1 January 2021 and 30 June 2025, 784,372 children aged 6–12 years underwent eye examinations at Salud Digna diagnostic clinics located throughout Mexico. The mean age of the study population was 9.7 years (SD = 1.91), with 421,168 (53.69%) females and 363,204 (46.31%) males. Among these children, 530,205 (67.6%) reported that they were not wearing glasses at the time of their examination. Table 1 presents a summary of the demographic and clinical characteristics of the study population.

### 3.1. Clinical Prevalence of Astigmatism and Astigmatic Amblyogenic Ametropias

**Figure 1:**
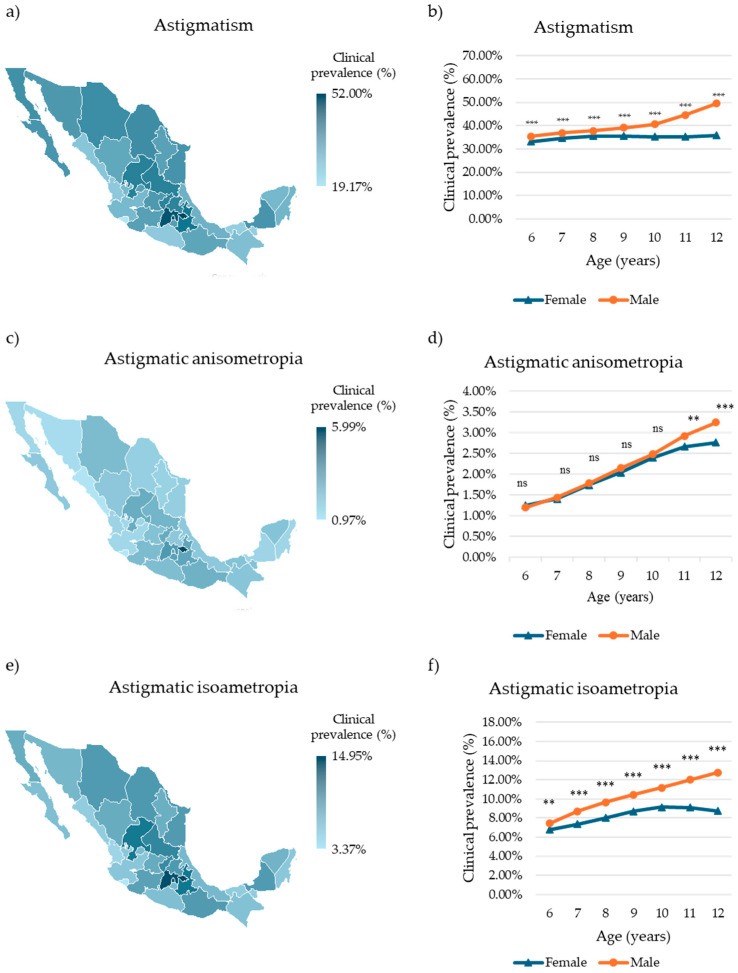
Clinical prevalence of astigmatism and astigmatic amblyogenic ametropia in Mexican children. (**a**) Choropleth map of the clinical prevalence of astigmatism in Mexican children by state. (**b**) Clinical prevalence of astigmatism in Mexican children by sex and age. (**c**) Choropleth map of the clinical prevalence of astigmatic amblyogenic anisometropia in Mexican children by state. (**d**) Clinical prevalence of astigmatic amblyogenic anisometropia in Mexican children by sex and age. (**e**) Choropleth map of the prevalence of astigmatic amblyogenic isoametropia in Mexican outpatient children by state. (**f**) Clinical prevalence of astigmatic amblyogenic isoametropia in Mexican children according to sex and age. Differences in age-specific prevalence between sexes were evaluated using the chi-squared (χ^2^) test. Significative differences: ns = nonsignificant, ** *p* < 0.01, *** *p* < 0.001.

Nationally, astigmatism was identified in 301,284 children, representing a clinical prevalence of 38.41% (95% confidence interval (CI): 38.30–38.52%). The highest clinical prevalence was observed in Tlaxcala, with 1403 affected children (52.00%, 95% CI: 50.17–53.83%), while the lowest was recorded in Colima, with 1069 affected children (19.17%, 95% CI: 18.15–20.23%) (Figure 1a, Supplementary Table S1). Age- and sex-specific analysis revealed the highest clinical prevalence in 12-year-old children (females: 37,939 cases, 35.92%, 95% CI: 35.63–36.21%; males: 40,941 cases, 49.49%, 95% CI: 49.15–49.83%) and the lowest in 6-year-olds (females: 9390 cases, 33.22%, 95% CI: 32.68–33.78%; males: 9361 cases, 35.43%, 95% CI: 34.85–36.01%) (Figure 1b, Supplementary Table S2). Across all age groups, males exhibited a significantly higher clinical prevalence than females (*p* < 0.0001).

Regarding astigmatic amblyogenic ametropia, 18,080 children were diagnosed with astigmatic anisometropia, corresponding to a national clinical prevalence of 2.31% (95% CI: 2.27–2.34%). Tlaxcala again showed the highest clinical prevalence (175 cases, 5.99%, 95% CI: 5.15–6.91%), whereas Colima had the lowest (54 cases, 0.97%, 95% CI: 0.73–1.26%) (Figure 1c, Supplementary Table S3). Age and sex trends mirrored those observed for general astigmatism, with the highest clinical prevalence in 12-year-olds (females: 2925 cases, 2.77%, 95% CI: 2.67–2.87%; males: 2693 cases, 3.26%, 95% CI: 3.14–3.38%) and the lowest in 6-year-olds (females: 354 cases, 1.25%, 95% CI: 1.13–1.39%; males: 318 cases, 1.20%, 95% CI: 1.08–1.34%) (Figure 1d, Supplementary Table S4). Statistically significant sex differences were observed in children aged 11 and 12 years (11 y: *p* < 0.01; 12 y: *p* < 0.0001), whereas no significant differences were found in younger age groups (*p* > 0.5).

Astigmatic isoametropia was the most clinically prevalent amblyogenic ametropia, affecting 75,169 children (9.58%, 95% CI: 9.52–9.65%). Tlaxcala again had the highest clinical prevalence (437 cases, 14.95%, 95% CI: 13.68–16.30%), whereas Colima had the lowest (337 cases, 3.37%, 95% CI: 2.91–3.88%) (Figure 1e, Supplementary Table S3). Among females, the highest clinical prevalence was observed in 10-year-olds (5655 cases, 9.13%, 95% CI: 8.91–9.36%), whereas among males, it was highest in 12-year-olds (10,560 cases, 12.76%, 95% CI: 12.54–12.99%). The lowest prevalence in both sexes was found in 6-year-olds (females: 1922 cases, 6.80%, 95% CI: 6.51–7.10%; males: 1968 cases, 7.45%, 95% CI: 7.13–7.77%) (Figure 1f, Supplementary Table S4). Across all age groups, males consistently exhibited higher clinical prevalence than females (6 y: *p* < 0.01; 7–12 y: *p* < 0.0001).

### 3.2. Clinical Prevalence of Myopia and Myopic Amblyogenic Ametropias

**Figure 2:**
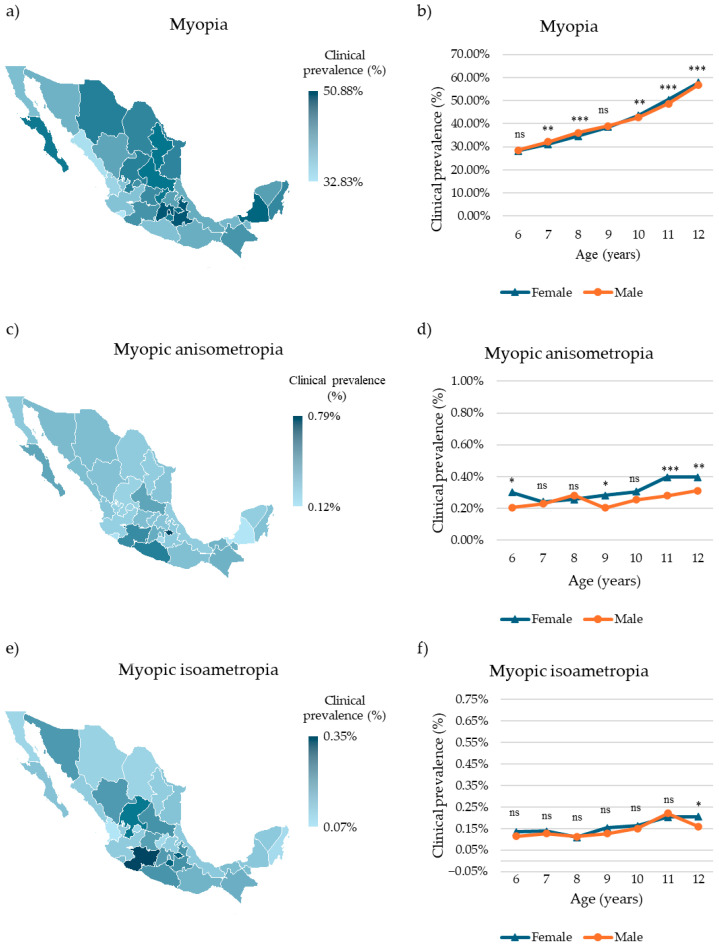
Clinical prevalence of myopia and myopic amblyogenic ametropias in Mexican children. (**a**) Choropleth map of the clinical prevalence of myopia in Mexican children by state. (**b**) Clinical prevalence of myopia in Mexican children by sex and age. (**c**) Choropleth map of the clinical prevalence of myopic amblyogenic anisometropia in Mexican children by state. (**d**) Clinical prevalence of myopic amblyogenic anisometropia in Mexican children by sex and age. (**e**) Choropleth map of the clinical prevalence of myopic amblyogenic isoametropia in Mexican children by state. (**f**) Clinical prevalence of myopic amblyogenic isoametropia in Mexican children by sex and age. Differences in age-specific prevalence between sexes were evaluated using the chi-squared (χ^2^) test. Significative differences: ns = nonsignificant, * *p* < 0.05, ** *p* < 0.01, *** *p* < 0.001.

Nationally, myopia was the most clinically prevalent RE, affecting 344,000 children (43.86%, 95% CI: 43.75–43.97%). The highest clinical prevalence was observed in Ciudad de Mexico, with 44,891 affected children (50.88%, 95% CI: 50.56–51.21%), whereas the lowest was recorded in Colima, with 1830 affected children (32.83%, 95% CI: 31.59–34.08%) (Figure 2a, Supplementary Table S1). Age- and sex-specific analysis revealed the highest clinical prevalence in 12-year-olds (females: 61,048 cases, 57.81%, 95% CI: 57.51–58.10%; males: 46,996 cases, 56.81%, 95% CI: 56.47–57.14%) and the lowest in 6-year-olds (females: 7987 cases, 28.26%, 95% CI: 27.74–28.79%; males: 7535 cases, 28.52%, 95% CI: 27.97–29.07%) (Figure 2b, Supplementary Table S2). Females aged 10 years and older exhibited a higher clinical prevalence than males (10 y: *p* < 0.01; 11–12 y: *p* < 0.0001), whereas males aged 7–8 years showed a higher clinical prevalence than females (7 y: *p* < 0.01; 8 y: *p* < 0.0001). No statistically significant differences were observed among the other age groups (*p* > 0.5).

Regarding myopic amblyogenic ametropias, 2331 children were diagnosed with myopic anisometropia, corresponding to a national clinical prevalence of 0.30% (95% CI: 0.29–0.31%). Tlaxcala exhibited the highest clinical prevalence (23 cases, 0.79%, 95% CI: 0.50–1.18%), whereas Campeche had the lowest (2 cases, 0.12%, 95% CI: 0.02–0.45%) (Figure 2c, Supplementary Table S3). Age and sex trends were consistent with general myopia patterns, with the highest clinical prevalence in 12-year-olds (females: 419 cases, 0.31%, 95% CI: 0.28–0.35%; males: 258 cases, 0.31%, 95% CI: 0.28–0.35%). The lowest clinical prevalence was observed in 7-year-old females and 6-year-old males (females: 105 cases, 0.24%, 95% CI: 0.20–0.29%; males: 54 cases, 0.20%, 95% CI: 0.15–0.27%) (Figure 2d; Supplementary Table S4). Males exhibited significantly higher clinical prevalence than females at 6, 9, 11, and 12 years of age (6 y and 9 y: *p* < 0.05; 11 y: *p* < 0.0001; 12 y: *p* < 0.01). No significant differences were observed among the other age groups (*p* > 0.5).

For myopic isoametropia, 1259 children were affected (0.16%, 95% CI: 0.15–0.17%). The highest clinical prevalence was found in Michoacán (48 cases, 0.35%, 95% CI: 0.26–0.46%), whereas the lowest was observed in Nayarit (6 cases, 0.07%, 95% CI: 0.03–0.15%) (Figure 2e, Supplementary Table S3). Among females, the highest clinical prevalence occurred in 12-year-olds (217 cases, 0.21%, 95% CI: 0.18–0.23%), whereas among males, it was highest in 11-year-olds (137 cases, 0.22%, 95% CI: 0.19–0.26%). The lowest clinical prevalence in both sexes was observed in 8-year-olds (females: 56 cases, 0.11%, 95% CI: 0.08–0.14%; males: 53 cases, 0.11%, 95% CI: 0.08–0.15%) (Figure 2f, Supplementary Table S4). Males exhibited a significantly higher clinical prevalence than females at 12-year-olds (*p* < 0.05), with no significant differences in other age groups (*p* > 0.5).

### 3.3. Clinical Prevalence of Hyperopia and Hyperopic Amblyogenic Ametropias

**Figure 3:**
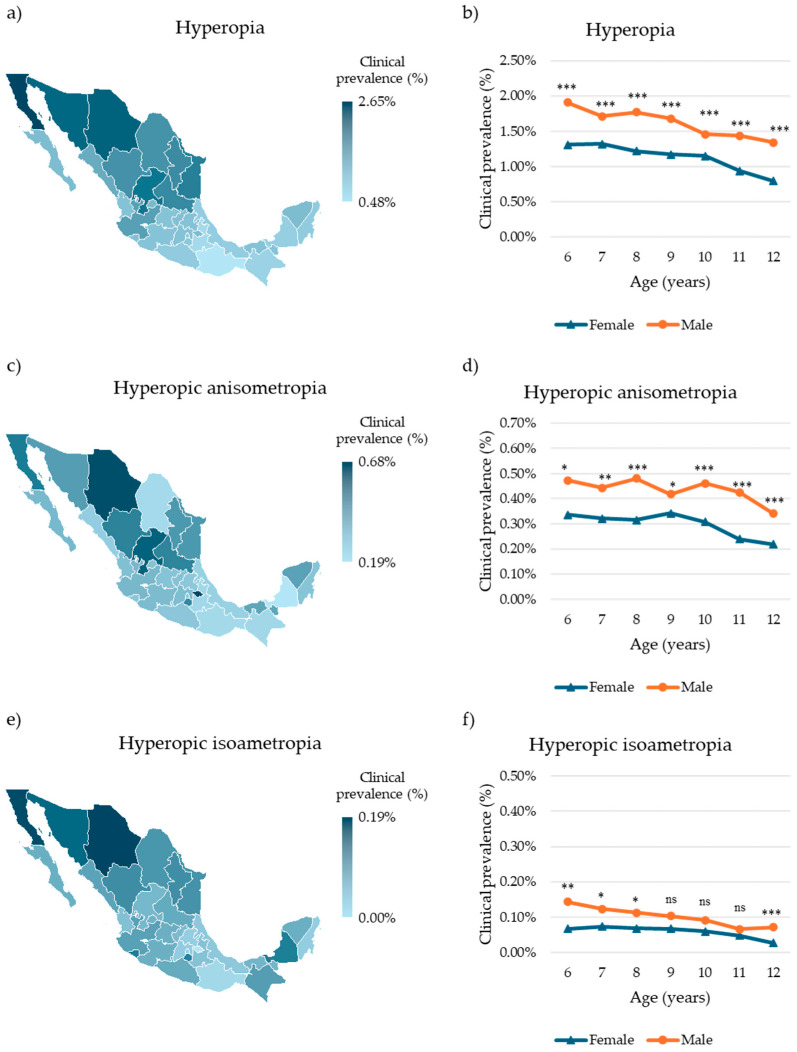
Clinical prevalence of hyperopia and hyperopic amblyogenic ametropias in Mexican children. (**a**) Choropleth map of the clinical prevalence of hyperopia in Mexican children by state. (**b**) Clinical prevalence of hyperopia in Mexican children by sex and age. (**c**) Choropleth map of the clinical prevalence of hyperopic amblyogenic anisometropia in Mexican children by state. (**d**) Clinical prevalence of hyperopic amblyogenic anisometropia in Mexican children by sex and age. (**e**) Choropleth map of the clinical prevalence of hyperopic amblyogenic isoametropia in Mexican children by state. (**f**) Clinical prevalence of hyperopic amblyogenic isoametropia in Mexican children by sex and age. Differences in age-specific prevalence between sexes were evaluated using the chi-squared (χ^2^) test. Significative differences: ns = nonsignificant, * *p* < 0.05, ** *p* < 0.01, *** *p* < 0.001.

Hyperopia was identified in 10,152 children, corresponding to a clinical prevalence of 1.29% (95% CI: 1.27–1.32%). The highest clinical prevalence was observed in Baja California (1282 cases, 2.65%, 95% CI: 2.51–2.80%); the lowest was recorded in Oaxaca (30 cases, 0.48%, 95% CI: 0.33–0.69%) (Figure 3a, Supplementary Table S1). Age- and sex-specific analysis revealed the highest clinical prevalence in 6-year-olds (females: 371 cases, 1.31%, 95% CI: 1.18–1.45%; males: 504 cases, 1.91%, 95% CI: 1.75–2.08%) and the lowest in 12-year-olds (females: 840 cases, 0.80%, 95% CI: 0.74–0.85%; males: 1110 cases, 1.34%, 95% CI: 1.26–1.42%) (Figure 3b, Supplementary Table S2). Across all age groups, males exhibited a significantly higher clinical prevalence than females (*p* < 0.0001).

Hyperopic anisometropia was diagnosed in 2727 children, representing a national clinical prevalence of 0.35% (95% CI: 0.33–0.36%). Tlaxcala showed the highest clinical prevalence (20 cases, 0.68%, 95% CI: 0.42–1.05%), whereas Campeche had the lowest (3 cases, 0.19%, 95% CI: 0.04–0.54%) (Figure 3c, Supplementary Table S3). Among females, the highest clinical prevalence was observed in 6-year-olds (258 cases, 0.34%, 95% CI: 0.27–0.41%), whereas among males, it was highest in 8-year-olds (225 cases, 0.48%, 95% CI: 0.42–0.55%). The lowest clinical prevalence in both sexes was found in 12-year-olds (females: 232 cases, 0.22%, 95% CI: 0.19–0.25%; males: 282 cases, 0.34%, 95% CI: 0.30–0.38%) (Figure 3d, Supplementary Table S4). Males exhibited significantly higher clinical prevalence than females across all age groups (6 y and 9 y: *p* < 0.05; 8 y, 10–12 y: *p* < 0.0001). No significant differences were observed among the other age groups (*p* > 0.5).

Hyperopic isoametropia was identified in 568 children (0.07%, 95% CI: 0.07–0.08%). The highest clinical prevalence was recorded in Chihuahua (41 cases, 0.19%, 95% CI: 0.13–0.25%), whereas no cases were observed in Tlaxcala (Figure 3e, Supplementary Table S3). Age- and sex-specific analysis showed the highest clinical prevalence in 6-year-olds (females: 19 cases, 0.07%, 95% CI: 0.04–0.10%; males: 38 cases, 0.14%, 95% CI: 0.10–0.20%), and the lowest in 12-year-olds (females: 28 cases, 0.03%, 95% CI: 0.02–0.04%; males: 59 cases, 0.07%, 95% CI: 0.05–0.09%) (Figure 3f, Supplementary Table S4). Males exhibited significantly higher clinical prevalence than females in children aged 6–8 and 12 years (6 y: *p* < 0.01; 7–8 y: *p* < 0.05; 12 y: *p* < 0.0001). No significant differences were observed among the other age groups (*p* > 0.5).

### 3.4. Refractive Amblyopia

After identifying children with RE and amblyogenic ametropia, we assessed whether these individuals had already developed amblyopia. Nationally, 38,722 children were diagnosed with refractive amblyopia, corresponding to a clinical prevalence of 4.94% (95% CI: 4.89–4.98%) (Supplementary Table S5). Of these, 19,141 (49.43%) were male, and 19,581 (50.57%) were female. The mean age was 9.7 years (SD = 1.93), and 17,899 (46.22%) did not wear glasses at the time of examination. Table 2 summarizes the demographic and clinical characteristics of this subpopulation of patients.

**Figure 4:**
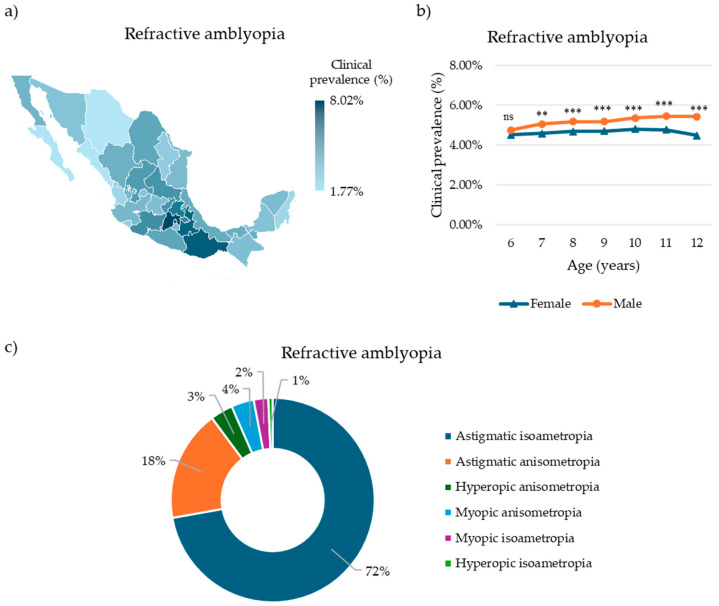
Clinical prevalence of refractive amblyopia in Mexican children. (**a**) Choropleth map of the clinical prevalence of refractive amblyopia in Mexican children by state. (**b**) Clinical prevalence of refractive amblyopia in Mexican children by sex and age. (**c**) Refractive amblyopia in Mexican children and its associated ametropia. Differences in age-specific prevalence between sexes were evaluated using the chi-squared (χ^2^) test. Significative differences: ns = nonsignificant, ** *p* < 0.01, *** *p* < 0.001.

In terms of geographical distribution, Estado de Mexico exhibited the highest clinical prevalence, with 11,800 affected children (8.02%, 95% CI: 7.89–8.17%), while Chihuahua reported the lowest (387 affected children, 1.77%, 95% CI: 1.60–1.96%) (Figure 4a, Supplementary Table S5).

Age- and sex-specific analyses revealed that the highest clinical prevalence among females occurred in 10-year-olds (2977 cases, 4.81%, 95% CI: 4.64–4.98%), whereas among males, it was highest in 12-year-olds (4503 cases, 5.44%, 95% CI: 5.29–5.60%). The lowest clinical prevalence in females was observed in 12-year-olds (4735 cases, 4.48%, 95% CI: 4.36–4.61%), and in males, in 6-year-olds (1257 cases, 4.76%, 95% CI: 4.50–5.02%) (Figure 4b, Supplementary Table S6). Males exhibited significantly higher clinical prevalence than females from ages 7 to 12 (7 y: *p* < 0.01; 8–12 y: *p* < 0.0001), with no significant differences observed in other age groups (*p* > 0.5).

Regarding the type of ametropia associated with refractive amblyopia, the majority of cases were linked to astigmatic isoametropia (27,954 cases, 72.19%), followed by astigmatic anisometropia (6835 cases, 17.65%), hyperopic anisometropia (1385 cases, 3.58%), myopic anisometropia (1385 cases, 3.58%), myopic isoametropia (897 cases, 2.32%), and hyperopic isoametropia (266 cases, 0.69%) (Figure 4c).

### 3.5. VI and Blindness Associated with RA

**Figure 5:**
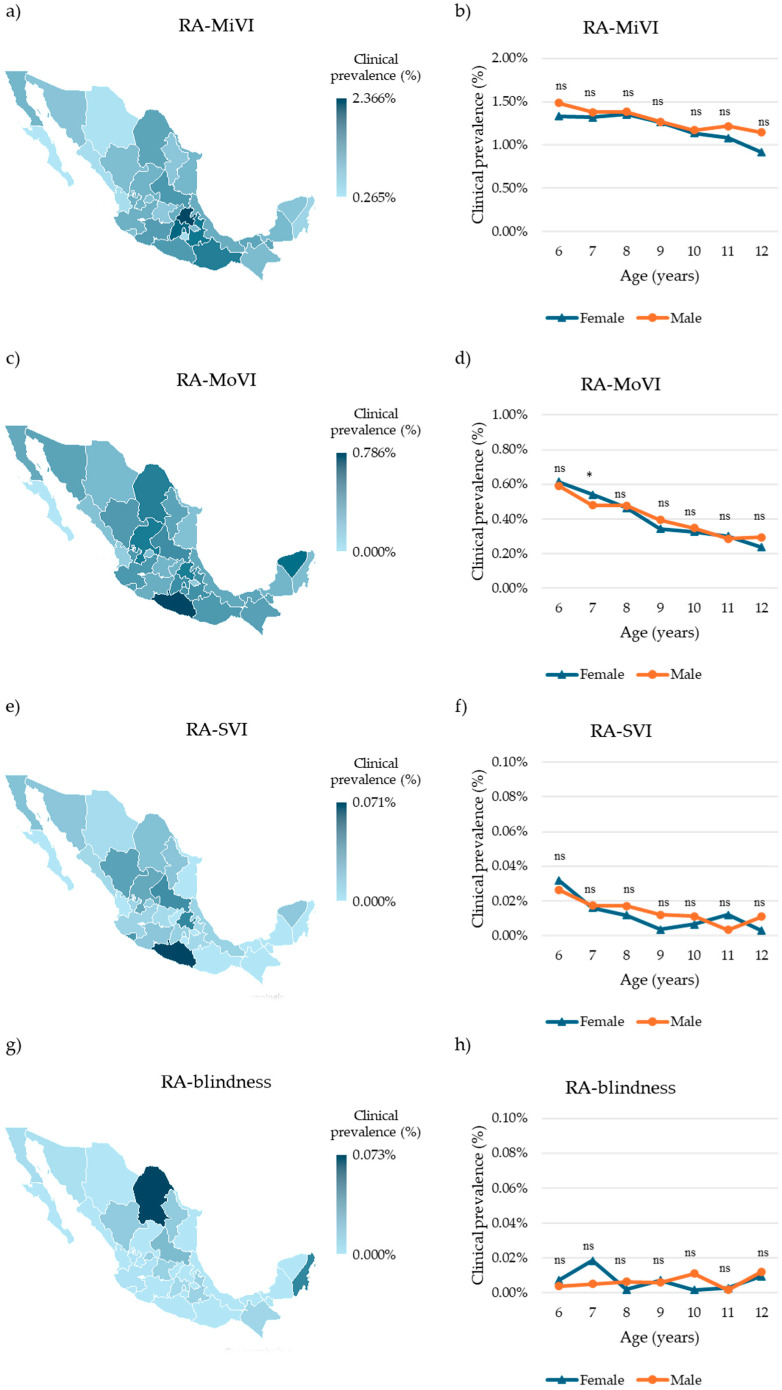
Visual impairment associated with refractive amblyopia in Mexican children. (**a**) Choropleth map of the clinical prevalence of mild visual impairment (MiVI) related to refractive amblyopia in Mexican children by state. (**b**) Clinical prevalence of MiVI related to refractive amblyopia in Mexican children by sex and age. (**c**) Choropleth map of the clinical prevalence of moderate visual impairment (MoVI) related to refractive amblyopia in Mexican children by state. (**d**) Clinical prevalence of MoVI related to refractive amblyopia in Mexican children by sex and age. (**e**) Choropleth map of the clinical prevalence of severe visual impairment (SVI) related to refractive amblyopia in Mexican children by state. (**f**) Clinical prevalence SVI) related to refractive amblyopia in Mexican children by sex and age. (**g**) Choropleth map of the clinical prevalence of blindness related to refractive amblyopia in Mexican children by state. (**h**) Clinical prevalence of blindness related to refractive amblyopia in Mexican children by sex and age. Differences in age-specific prevalence between sexes were evaluated using the chi-squared (χ^2^) test. Abbreviatures RA = Refractive amblyopia, MiVI = Mild visual impairment, MoVI = Moderate visual impairment, SVI = Severe visual impairment, Significative differences: ns = nonsignificant, * *p* < 0.05.

To evaluate the functional impact of refractive amblyopia, we analyzed the visual acuity data of affected children. Mild visual impairment (MiVI) related to RA was observed in 9411 children (1.20%, 95% CI: 1.18–1.22%). The highest clinical prevalence was recorded in Hidalgo (166 cases, 2.37%, 95% CI: 2.02–2.75%), whereas the lowest was in Baja California Sur (4 cases, 0.27%, 95% CI: 0.07–0.68%) (Figure 5a, Supplementary Table S7). Age- and sex-specific analyses showed the highest clinical prevalence in 8-year-old females (692 cases, 1.36%, 95% CI: 1.26–1.46%) and 6-year-old males (393 cases, 1.49%, 95% CI: 1.34–1.64%). The lowest clinical prevalence was observed in 12-year-olds of both sexes (females: 968 cases, 0.92%, 95% CI: 0.86–0.98%; males: 948 cases, 1.15%, 95% CI: 1.07–1.22%) (Figure 5b, Supplementary Table S8). No statistically significant differences were found across age groups (*p* > 0.5).

**Figure 6:**
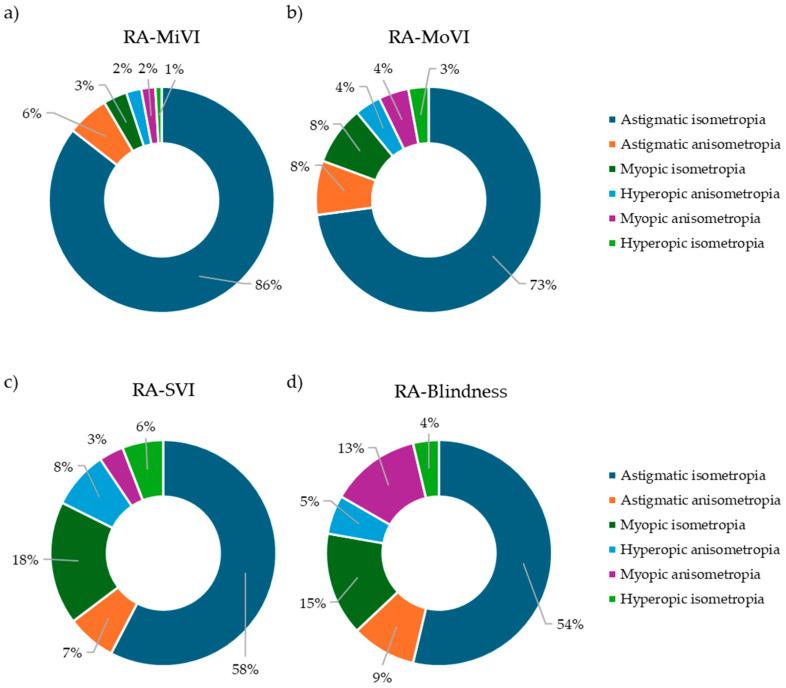
Visual impairment associated with refractive amblyopia and its related ametropia in Mexican children. (**a**) Mild visual impairment associated with refractive amblyopia and related ametropy in Mexican children. (**b**) Moderate visual impairment associated with refractive amblyopia and related ametropy in Mexican children. (**c**) Severe visual impairment associated with refractive amblyopia and related ametropy in Mexican children. (**d**) Blindness associated with refractive amblyopia and its related ametropy in Mexican outpatient children. Abbreviatures RA = Refractive amblyopia, MiVI = Mild visual impairment, MoVI = Moderate visual impairment, SVI = Severe visual impairment.

Among children with MiVI, the majority were associated with astigmatic isoametropia (8050 cases, 85.54%), followed by astigmatic anisometropia (564 cases, 5.99%), myopic isoametropia (319 cases, 3.39%), hyperopic anisometropia (203 cases, 2.16%), myopic anisometropia (189 cases, 2.01%), and hyperopic isoametropia (86 cases, 0.91%) (Figure 6a).

Moderate VI (MoVI) related to refractive amblyopia was identified in 2896 children (0.37%, 95% CI: 0.36–0.38%). Guerrero had the highest clinical prevalence (44 cases, 0.79%, 95% CI: 0.57–1.05%), while no cases were reported in Baja California Sur (Figure 5c, Supplementary Table S7). The highest clinical prevalence was observed in 6-year-olds (females: 174 cases, 0.62%, 95% CI: 0.53–0.71%; males: 156 cases, 0.59%, 95% CI: 0.50–0.69%), and the lowest in 12-year-olds (females: 0.24%, 95% CI: 0.21–0.27%; males: 0.29%, 95% CI: 0.26–0.33%) (Figure 5d, Supplementary Table S8). A statistically significant difference was observed in 7-year-old females (*p* < 0.05); however, no other age group showed significance (*p* > 0.5).

Among MoVI cases, astigmatic isoametropia was again the most common cause (2110 cases, 72.86%), followed by myopic isoametropia (244 cases, 8.43%), astigmatic anisometropia (225 cases, 7.77%), myopic anisometropia (125 cases, 4.32%), hyperopic anisometropia (108 cases, 3.73%), and hyperopic isoametropia (84 cases, 2.90%) (Figure 6b).

Severe VI (SVI) related to RA was observed in 85 children (0.01%, 95% CI: 0.01–0.01%). Guerrero had the highest clinical prevalence (4 cases, 0.07%, 95% CI: 0.02–0.18%), while no cases were reported in Baja California Sur, Campeche, Chiapas, Morelos, Nayarit, Oaxaca, Querétaro, Quintana Roo, Tabasco, Tamaulipas, and Tlaxcala (Figure 5e, Supplementary Table S7). The highest clinical prevalence was found in 6-year-olds (females: 9 cases, 0.03%, 95% CI: 0.01–0.06%; males: 6 cases, 0.03%, 95% CI: 0.01–0.05%). The lowest clinical prevalence was observed in 9-year-old females and 11-year-old males (two cases each, 0.00%, 95% CI: 0.00–0.01%) (Figure 5f, Supplementary Table S8). No statistically significant differences were found across age groups (*p* > 0.5).

Among SVI cases, astigmatic isoametropia was the leading cause (49 cases, 57.65%), followed by myopic isoametropia (15 cases, 17.65%), hyperopic anisometropia (7 cases, 8.24%), astigmatic anisometropia (6 cases, 7.06%), hyperopic isoametropia (5 cases, 5.88%), and myopic anisometropia (3 cases, 3.53%) (Figure 6c).

Blindness related to refractive amblyopia was identified in 54 children (0.01%, 95% CI: 0.01–0.01%). Coahuila had the highest clinical prevalence (13 cases, 0.07%, 95% CI: 0.04–0.12%), whereas 18 states reported no cases (Figure 5g, Supplementary Table S7). The highest clinical prevalence in females was observed in 7-year-olds (8 cases, 0.02%, 95% CI: 0.01–0.04%), and in males, among 12-year-olds (10 cases, 0.01%, 95% CI: 0.01–0.02%); however, the lowest clinical prevalence was found in 8-year-old females and 11-year-old males (one case each, 0.00%, 95% CI: 0.00–0.01%) (Figure 5h, Supplementary Table S8). No statistically significant differences were observed across age groups (*p* > 0.5).

Among blindness cases, astigmatic isoametropia was the most frequent cause (29 cases, 53.70%), followed by myopic isoametropia (8 cases, 14.81%), myopic anisometropia (7 cases, 12.96%), astigmatic anisometropia (5 cases, 9.26%), hyperopic anisometropia (3 cases, 5.56%), and hyperopic isoametropia (2 cases, 3.70%) (Figure 6d).

## 4. Discussion

In Mexico, data on REs, refractive amblyopia, and associated VI in children remain scarce. This study analyzed a large outpatient pediatric population across all 32 Mexican states, providing a comprehensive national overview. Our aim was to characterize the epidemiological profiles of RE, RA, and related VI in outpatient children. We expect that our findings will inform public health strategies focused on pediatric vision care, including screening, management, and treatment, to mitigate both academic and socio-economic consequences.

Myopia was the most clinical prevalent RE in our cohort (43.86%), followed by astigmatism (38.41%) and hyperopia (1.29%). The prevalence of myopia and astigmatism exceeded previously reported rates in Mexican children [46,47,48], whereas hyperopia was less frequent than in earlier studies [46,47,48]. Compared to international data, our rates of myopia and astigmatism were higher than those in some reports [54] but lower than those observed by Hönekopp et al. [55] in German children. The prevalence of hyperopia in our study was also lower than the international estimates [54,56]. When placed in a broader context, our findings align with global trends documented in large-scale studies such as the Refractive Error Study in Children (RESC), which reported myopia prevalence ranging from 1.2% in rural Nepal to over 36% in urban China, additionally underscoring the role of urbanization and near-work behaviors [50]. These comparisons highlight the multifactorial nature of refractive error epidemiology and reinforce the need for region-specific strategies based on global evidence.

Across all age groups, males consistently exhibited higher prevalence rates of astigmatism, myopia, and hyperopia than females, with statistically significant differences observed in nearly all age strata. These findings indicate an association between sex and the prevalence of REs during childhood, potentially influenced by behavioral, environmental and biological factors. Further research is warranted to explore the underlying mechanisms driving these disparities and determine whether sex-specific screening or intervention strategies are beneficial.

Comparative data on amblyogenic ametropia are limited because of the lack of prior research in this area. To the best of our knowledge, this is the first nationwide report from Mexico addressing the amblyogenic potential of REs. We found that myopic and hyperopic ametropias had prevalences below 0.5%, whereas astigmatic ametropias exceeded 2%, with astigmatic isoametropia reaching 9.58%. These findings are consistent with the hypothesis that astigmatic ametropias are the primary contributors to refractive amblyopia and refractive amblyopia related VI in Mexican children.

Regarding amblyopia, we report a national refractive amblyopia prevalence of 4.94%, consistent with previous estimates of amblyopia in Mexico (2–5%) [44,45] and broader international estimates [33,34,35,36,37]. Sex-based analysis revealed a higher prevalence in males, aligning with global findings of Hu et al. [32].

Our estimates of VI associated with refractive amblyopia were lower than those reported in previous studies of Mexican schoolchildren [47] and broader Latin American populations [57]. The interpretation of visual impairment associated with refractive amblyopia should be approached cautiously, as we cannot rule out the contribution of uncorrected refractive errors, since 46.22% of children diagnosed with refractive amblyopia in this cohort were not wearing corrective lenses at the time of examination.

Discrepancies in prevalence may stem from differences in sample size, data collection methods, definitions of REs (particularly hyperopia), and genetic or ethnic variability, complicating direct comparisons with previous research.

It is important to note that our study population consisted of self-selected outpatients seeking optical services, in contrast to the population-based screening studies. This may have led to an overrepresentation of certain conditions in our sample, as we presented estimations of clinical prevalence instead of population prevalence.

Geographic variations in refractive error and amblyopia prevalence likely reflect a multifactorial interplay of genetic, environmental, and socioeconomic determinants. Northern states, where hyperopia was more frequent, have a higher proportion of European ancestry, consistent with reports linking hyperopia to this genetic background [56]. Conversely, the central and southern regions, which exhibit elevated rates of myopia and astigmatism, are characterized by greater urbanization and higher educational intensity, factors associated with increased near-work and reduced outdoor exposure, both of which are established risk factors for myopia progression [2]. Additionally, disparities in access to eye care and corrective lenses may exacerbate the prevalence of amblyopia in these areas. These findings underscore the need for region-specific strategies that consider ancestry, lifestyle, and healthcare accessibility when designing screening and preventive programs.

Notably, the geographical distribution of VI related to refractive amblyopia showed a unique pattern. States such as Guerrero and Coahuila, which had relatively low RE and refractive amblyopia prevalence, exhibited the highest rates of advanced VI. This highlights the need for targeted research on the biological and sociocultural determinants driving these disparities.

Given these findings, there is an urgent need to implement and strengthen school-based screening programs for REs and amblyopia in the future. Implementing school-based vision screening is widely recognized as a cost-effective strategy for the early detection of RE and amblyopia. The WHO’s World Report on Vision and its implementation handbooks recommend integrating screening into existing school health programs to reduce the long-term costs associated with untreated visual impairment and improve educational outcomes [52,58,59].

A practical framework includes training teachers for initial screening, establishing referral pathways to optometrists, subsidizing corrective lenses through partnerships, and implementing follow-up systems to ensure compliance [60,61]. These strategies align with the WHO and PAHO guidelines, emphasizing early detection, equitable access, and integration into primary healthcare systems. Although schools provide the most effective platform for detecting and addressing common vision problems, community-based screening programs remain essential to reach children with complex conditions that hinder regular school attendance [62].

Finally, it is concerning that among the 5678 children identified with refractive amblyopia who were not wearing glasses at the time of examination, none acquired corrective lenses afterward. This indicates that their condition remained untreated, highlighting the need for improved access to vision care and follow-up services to limit visual affection. In this context, it is essential to enhance ophthalmic human resources in Mexico, as the current ratio of 43 ophthalmologists and 47 optometrists per million people is insufficient to adequately serve the entire population [49].

### Strengths and Limitations of the Study

The strength of this study lies in its large nationwide sample covering all 32 Mexican states. Eye examinations were conducted by certified optometrists across all clinics following a standardized protocol. However, all measurements were obtained without cycloplegia, which is known to influence the refractive assessment.

Non-cycloplegic refraction is prone to overestimating myopia and underestimating hyperopia [63,64], thereby introducing potential bias in prevalence estimates for pediatric populations. In this regard, non-cycloplegic spherical equivalent (SE) has been reported to measure 0.65 ± 1.04 diopters (D) more myopic than cycloplegic SE, with differences of at least 1.00 D of more myopic SE being more likely to be observed in Hispanic individuals. Due to the nature of the electronic health records analyzed in this study, cycloplegic data could not be collected.

In the context of refractive amblyopia, it is important to consider that strabismus and other ocular pathologies not documented in electronic health records may lead to the misclassification of non-refractive amblyopia as refractive amblyopia, potentially inflating the estimates of refractive amblyopia prevalence.

Moreover, sensitivity analyses using different BCVA thresholds, such as 20/25 or 20/40, were not performed because this study was designed as a descriptive epidemiological analysis based on standardized electronic health records data. The classification criteria (BCVA ≤ 20/30) were established beforehand, as outlined in the methods section, and the retrospective nature of the dataset prevented reclassification without risking consistency and comparability within the cohort. Future studies will explore alternative thresholds and more detailed definitions.

While our findings revealed associations between refractive error patterns and certain demographic or geographic factors, these relationships should not be interpreted as causal due to the retrospective nature of this study. The observed differences may reflect underlying behavioral, environmental, or socioeconomic influences; however, further longitudinal research is required to confirm these hypotheses. Similarly, any discussion of potential social or developmental consequences of refractive errors should be framed as correlational rather than causal.

Moreover, multivariable regression analyses were not conducted because this study was intended to be a descriptive epidemiological report utilizing standardized electronic health records data, and further modeling was outside the scope of this research. As a result, the findings only reflect unadjusted associations, and prevalence estimates should not be seen as evidence of independent risk factors. Future research will include multivariable models to gain a deeper understanding of these relationships.

Additionally, the cohort consisted of outpatients who voluntarily sought diagnostic services at Salud Digna, creating a self-selected sample that included individuals from diverse socioeconomic backgrounds. This selection process may lead to higher observed rates of refractive errors and amblyopia than those in population-based studies, as these patients are more likely to present with visual symptoms. Although this bias is partially offset by the large dataset enabling detailed subgroup analyses, the findings should be interpreted within the context of an outpatient population rather than the general population.

Hence, our results cannot be interpreted as general population prevalence for policy making; nevertheless, they can set the basis for further research in Mexican population.

## 5. Conclusions

Our study highlights the burden of REs and refractive amblyopia in Mexican children and their impact on visual capacity in this population. Given that amblyopia is the leading cause of VI in children and young adults, screening is crucial for its early detection. We hope that our data provide a basis for further studies in children, leading to appropriate multisectoral cooperation in Mexico and countries with similar cultural and ancestral features. This collaboration between the public and private sectors is necessary to develop strategies to improve access to visual health services, increase the number of eye care professionals, and promote early screening, treatment, and management.

Such efforts are vital for reducing the lifelong clinical and social impacts of REs and amblyopia. Moreover, it is advisable to employ longitudinal studies and population-based surveys utilizing standardized methodologies to monitor the progression of REs, amblyopia, and VI. These approaches are essential for evaluating the effectiveness of interventions, identifying emerging risk factors, and informing future policies.

## Figures and Tables

**Table 1:** Demographic characteristics of the children outpatient included in this study.

Characteristic	n	%
**Sex**		
Male	363,204	46.31%
Female	421,168	53.69%
**Age**	9.7 (SD = 1.91)	
6	54,685	6.97%
7	83,865	10.69%
8	97,836	12.47%
9	106,524	13.58%
10	116,382	14.84%
11	136,741	17.43%
12	188,339	24.01%
**Use of glasses**		
Yes	251,414	32.05%
No	530,205	67.60%
Missed	2753	0.35%

**Table 2:** Demographic characteristics of the children outpatients included in this study who were identified with refractive amblyopia.

Characteristic	n	%
**Sex**		
Male	19,141	49.43%
Female	19,581	50.57%
**Age**	9.7 (SD = 1.93)	
6	2535	6.55%
7	4036	10.42%
8	4824	12.46%
9	5249	13.56%
10	5899	15.23%
11	6941	17.93%
12	9238	23.86%
**Use of glasses**		
Yes	20,636	53.29%
Glasses acquisition		
Yes	15,776	76.45%
No	4860	23.55%
No	17,899	46.22%
Glasses acquisition		
Yes	12,221	68.28%
No	5678	31.72%
Missed	187	0.48%
Glasses acquisition		
Yes	132	70.59%
No	55	29.41%

## Data Availability

The data supporting the findings of this study are available from the corresponding author upon reasonable request. Owing to legal and ethical restrictions under the Mexican Federal Law on Protection of Personal Data Held by Private Parties, access to the data is limited to ensure the privacy and confidentiality of the study participants. Requests will be evaluated according to institutional policies and applicable regulations.

## Supplementary Materials

The following supporting information can be downloaded at https://www.mdpi.com/article/10.3390/children12121641/s1, Table S1: Prevalence of astigmatism, myopia and hyperopia in Mexican outpatients by state; Table S2: Prevalence of astigmatism, myopia and hyperopia in children Mexican outpatients by sex and age; Table S3: Prevalence of amblyogenic ametropias in Mexican children outpatients by state; Table S4: Prevalence of amblyogenic ametropias in Mexican children outpatients by sex and age; Table S5: Prevalence of refractive amblyopia in Mexican children outpatients by state; Table S6: Prevalence of refractive amblyopia in Mexican children outpatients by sex and age; Table S7: Prevalence of refractive amblyopia related visual impairment in Mexican children outpatients by state; Table S8: Prevalence of refractive amblyopia related visual impairment in Mexican children outpatients by sex and age.

## Abbreviations

The following abbreviations are used in this manuscript:

RE	Refractive error
VI	Visual impairment
SE	Spherical equivalent
D	Diopters
WHO	World Health Organization
CI	Confidence interval
MiVI	Mild visual impairment
MoVI	Moderate visual impairment
SVI	Severe visual impairment
